# Identification of an Intermediate Step in Foamy Virus Fusion

**DOI:** 10.3390/v12121472

**Published:** 2020-12-21

**Authors:** Aurélie Dupont, Ivo M. Glück, Dorothee Ponti, Kristin Stirnnagel, Sylvia Hütter, Florian Perrotton, Nicole Stanke, Stefanie Richter, Dirk Lindemann, Don C. Lamb

**Affiliations:** 1 Department of Chemistry, Ludwig Maximilians-Universität München, Butenandtstraße 5-13, 81377 München, Germany; aurelie.dupont@univ-grenoble-alpes.fr (A.D.); Ivo.Glueck@cup.lmu.de (I.M.G.); dorothee_ponti@gmx.de (D.P.); florian.perrotton@ens-lyon.fr (F.P.); 2 Center for Nano Science (CENS), Ludwig Maximilians-Universität München, Butenandtstraße 5-13, 81377 München, Germany; 3 LIPhy, University Grenoble Alpes, CNRS, F-38000 Grenoble, France; 4 Medical Faculty “Carl Gustav Carus”, Institute of Virology, Technische Universität Dresden, Fetscherstr. 74, 01307 Dresden, Germany; kstirnnagel@web.de (K.S.); sylvia.huetter86@gmail.com (S.H.); nicole.stanke@tu-dresden.de (N.S.); stefanie.richter4@tu-dresden.de (S.R.); 5 CRTD/DFG-Center for Regenerative Therapies, Technische Universität Dresden, Fetscherstr. 105, 01307 Dresden, Germany; 6 Nanosystems Initiative München (NIM), Ludwig Maximilians-Universität München, Butenandtstraße 5-13, 81377 München, Germany; 7 Center for Integrated Protein Science (CIPSM), Ludwig Maximilians-Universität München, Butenandtstraße 5-13, 81377 München, Germany

**Keywords:** foamy virus, viral fusion, retrovirus, envelope glycoprotein, capsid, single virus tracking, fluorescence live cell imaging, spinning disk confocal microscopy

## Abstract

Viral glycoprotein-mediated membrane fusion is an essential step for productive infection of host cells by enveloped viruses; however, due to its rarity and challenges in detection, little is known about the details of fusion events at the single particle level. Here, we have developed dual-color foamy viruses (FVs) composed of eGFP-tagged prototype FV (PFV) Gag and mCherry-tagged Env of either PFV or macaque simian FV (SFVmac) origin that have been optimized for detection of the fusion process. Using our recently developed tracking imaging correlation (TrIC) analysis, we were able to detect the fusion process for both PFV and SFVmac Env containing virions. PFV Env-mediated fusion was observed both at the plasma membrane as well as from endosomes, whereas SFVmac Env-mediated fusion was only observed from endosomes. PFV Env-mediated fusion was observed to happen more often and more rapidly than as for SFVmac Env. Strikingly, using the TrIC method, we detected a novel intermediate state where the envelope and capsids are still tethered but separated by up to 400 nm before final separation of Env and Gag occurred.

## 1. Introduction

Several genera of spumaviruses constitute the Spumaretrovirinae subfamily of retroviruses. Spumaviruses, also known as foamy viruses (FVs), are endemic to a wide range of vertebrates [1,2]. They are thought to be the oldest retroviruses, having emerged >450 million years ago coinciding with the origin of jawed vertebrates [3] and display a remarkably stable co-speciation with their hosts. Their largest genus, *Simiispumavirus*, combines all known FV species identified in different non-human primates of new- and old-world monkey, and ape origin. Prototype FV (PFV) is the best studied primate FV isolate. It was the first retrovirus discovered in humans, originally described as human FV, but was later recognized as being derived from a zoonotic transmission of a chimpanzee FV to man [4,5]. A hallmark of FVs, setting them apart from all other retroviruses, is their apparent apathogenicity, not only in their natural hosts but also in zoonotically infected humans. Although the FV genome structure is typical for a complex orthoretrovirus, FVs were grouped into a separate subfamily because research showed that their replication strategy deviates in several aspects from that of all other retroviruses [2]. Examples of special features of FVs in comparison to orthoretroviruses are a strictly viral glycoprotein (Env)-dependent particle egress that involves budding predominantly at intracellular membranes; a release of capsid-less, Env-containing subviral particles, and an extremely broad tropism that employs heparin sulfate attachment and currently unknown specific entry receptor(s).

**Figure 1:**
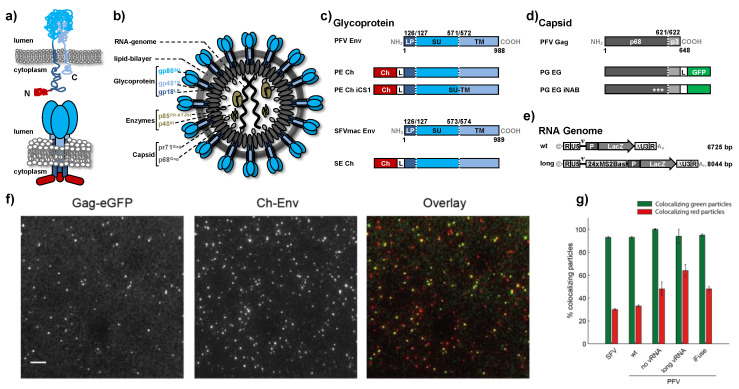
Characterization of the virus particles used in this study. (**a**) Schematic illustration of the prototype foamy viruses (PFV) Env membrane topology at the state of the precursor (upper graphic) and oligomeric, mature glycoprotein complex (lower graphic). Color coding of the individual domains or subunits is indicated in panels (**b**–**e**). (**b**) Schematic representation of the PFV particle structure. pr: precursor protein; p: protein; gp: glycoprotein; gp18^LP^: Env leader peptide subunit; gp80^SU^: Env surface subunit; gp48^TM^: Env transmembrane subunit; p85^PR-RT-RH^: Pol protease-reverse-transcriptase-RNaseH subunit; p40^IN^: Pol integrase subunit; pr71^Gag^: Gag precursor; p68^Gag^: Gag p68 subunit. (**c**–**e**) Schematic outline of the PFV Gag, PFV, or macaque simian foamy virus (SFVmac) Env, and PFV vector genome variants. Graphical illustration of the PFV Gag and PFV or SFVmac Env protein structure as well as the PFV viral genome structure of the different variants employed in this study. Numbers indicate the amino acid positions and the subunit processing sites within the translated precursor proteins are indicated by dashed lines. The viral RNA genome sizes are given in base pairs. NH_2_: N-terminus; COOH: C-terminus; LP: Env leader peptide domain; SU: Env surface domain; TM: Env transmembrane domain; Ch: mCherry open reading frame; L: glycine-serine linker peptide; p68: Gag p68 domain; p3: Gag p3 domain; GFP: eGFP open reading frame; ***: Gag glycine-arginine box deletions; R: long terminal repeat (LTR) repeat regions; U5: LTR unique 5′ region; ∆U3: enhancer–promoter deleted LTR unique 3′ region; ©: Cap structure; A_n_: poly A tail. (**f**) Virus particles spotted on a glass slide and recorded under widefield illumination using alternating laser excitation. Scale bar 10 µm. (**g**) Quantification of the particles used in this study according to their colocalization in both channels. Colocalizing green particles: Green particles colocalizing with a red particle signal; Colocalizing red particles: Red particles colocalizing with a green particle signal. Error bars show standard error of the mean determined from three fields of view.

FV structural protein synthesis and virion morphogenesis is also characterized by several special features that make these viruses an interesting tool for single particle tracing analysis using fluorescently labeled virions. First, FV Env biosynthesis is unique amongst retroviruses as the glycoprotein precursor is not cotranslationally processed by cellular signal peptidase, removing the N-terminal signal peptide, during translation into the secretory pathway [6,7,8,9]. Instead, a full-length Env precursor is translated and initially adopts a membrane topology with both N- and C-terminus located in the cytoplasm (Figure 1a) [7,8]. Only during cell surface transport is the Env precursor postranslationally modified and proteolytically processed by furin or furin-like proteases into the mature leader peptide (gp18^LP^), surface (gp80^SU^) and transmembrane (gp48^TM^) subunits [6,9]. All three subunits are integral components of a heterotrimeric glycoprotein complex in released PFV virions with leader peptide (LP) and transmembrane (TM) subunits adopting a type II and type I membrane topology, respectively (Figure 1a,b). The extracellular surface (SU) subunit appears to be associated with LP and TM subunit through non-covalent interactions. On released FV virions the mature Env glycoprotein complex forms prominent spike structures that are organized in an elaborate network of interlocked hexagons [10,11].

FV virion release, unlike orthoretroviruses, is characterized by a strict dependence of Gag and Env co-expression as the FV Gag proteins lack membrane targeting or membrane association signals [12,13]. This Gag feature results in an accumulation of naked preassembled capsid at the centrosome and prevents an orthoretroviral-like release of virus-like particle release in the absence of Env coexpression. The Env-dependence of FV budding and release is the consequence of a direct interaction of the N-terminus of Gag in capsids preassembled at the centrosome and the N-terminal cytoplasmic domain of the Env LP subunit [7,8,14,15]. The active participation of FV glycoproteins in virion morphogenesis is also emphasized by their ability to induce the release of capsid-less subviral particles, similar to what is observed for the hepatitis B virus S protein [16,17].

Target cell entry of most FVs is thought to require endocytosis and involve a viral glycoprotein-mediated fusion of viral and cellular lipid membranes that is predominantly dependent on low pH [18,19]. Only PFV Env was previously reported to possess significant fusion activity at neutral pH, which is in line with the observation that PFV Env containing retroviral particles can fuse with target cells at the plasma membrane or from within endocytic vesicles.

Most of what has been learned regarding virus fusion has been obtained using bulk experiments. However, direct information regarding the kinetics and details of the fusion process are missing. With the development of single virus tracing (SVT) techniques [20,21], it is now possible to follow the viral–host cell interactions of a single virion and thereby elucidate new details regarding the fusion processes. Pioneering experiments revealed that the adeno-associated virus runs through several stages of motion, each characterized by distinct diffusion characteristics during the infection pathway into living cells [21]. Groundbreaking work on influenza viruses determined the kinetics of hemifusion and content mixing using an in vitro system [22] and characterized the infection process in living cells [23,24]. Several studies applied SVT to elucidate details of the fusion process of Simian virus 40 [25,26,27] as well as the one of the human polyoma virus [28] and the echovirus I [29].

Here, we use dual-color FV constructs and our recently developed tracking image correlation (TrIC) approach [30] to visualize the fusion event in both PFV (SFVpsc) and SFVmac (SFVmcy). The fusion of PFV was observed both at the plasma membrane as well as from endosomes and was observed to happen on the timescale of 10 to 20 min. In contrast, fusion of SFVmac was only observed from endosomes and occurred on timescales longer than was measured for the fusion of PFV in endosomes. This is consistent with the higher fusogenicity of PFV Env at neutral pH as observed previously [18,19]. Both types of viruses exhibited an intermediate state where the fluorescence signal from the envelope and capsid are separated by 100 to 400 nm but are still tethered together. This intermediate persists on the timescale of 6 to 11 min and is independent of the properties of the packaged RNA.

## 2. Materials and Methods

### 2.1. Expression Constructs and Virus Preparation

The 4-component PFV vector system consisting of the packaging plasmids containing either authentic or expression-optimized ORF encoding wildtype or mutant PFV (SFVpsc) Gag, PFV Pol, PFV Env or SFVmac (SFVmcy) Env and PFV transfer vectors encoding a lacZ reporter gene was described previously [18,31,32]. In addition to fluorescent protein tagged wild-type constructs, the following mutant variants were used for this study: PFV Env: pcoPE iCS1 (iFuse) encoding a surface–transmembrane subunit furin cleavage site variant by R571T mutation [18]; PFV Gag: pcoPG4 iNAB1 (iNAB) encoding a nucleic acid binding deficient variant by deletion of glycine-arginine rich (GR) boxes I to III [33]; and PFV Pol: pcoPP2 or pcziPol iRT encoding a variant with catalytically inactive reverse transcriptase by YVDD312–315GAAA mutation. A schematic outline of FV Gag, Env packaging and PFV transfer vector variants are shown in Figure 1c–e.

The dual-colored FV particles (Gag-GFP and mCherry-Env) were prepared and concentrated as explained previously [18]. Briefly, most PFV Env (PE) containing supernatants were generated by cotransfection of 293T cells with the transfer vector pMD11 (wt vgRNA) or puc2MD11 MS2Bas (long vgRNA) and expression-optimized packaging plasmids encoding PFV Pol (pcoPP2), PFV Env (pcoPE Ch, pcoPE Ch iCS1) and PFV Gag (pcoPG4, pcoPG4 CeGFP, pcoPG4 CeGFP iNAB1) at a ratio of 28:2:1:4. SFVmac Env (SE) and some PE containing supernatants were produced by cotransfection of the transfer vector pMD11 and packaging plasmids encoding PFV Gag (pcziGag-CeGFP), PFV Pol (pcziPol iRT), and PFV Env (pczPE iCS2) or SFVmcy (SFVmac) Env (pciSE Ch) at a ratio of 1:1:1:1. Cell-free viral supernatants were harvested 48 h post transfection and viral particles concentrated by ultracentrifugation or ultrafiltration and aliquots snap-frozen on dry ice and stored at −80 °C until further use.

### 2.2. Spinning Disk Confocal Microscope

**Figure A1:**
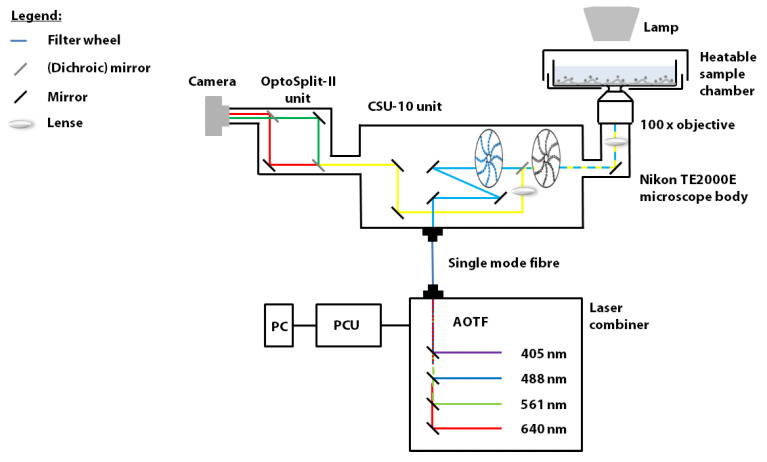
Spinning disk confocal microscope setup. A modified confocal spinning disk setup based on an Andor Revolution system was used. Continues wave laser lines of 405 nm, 488 nm, 561 nm and 640 nm wavelength built into the Andor Laser Combiner were directed into a single mode fiber. Continues wave laser lines of 445 nm, 514 nm, 594 nm and 685 nm were combined with the appropriate dichroic mirrors and directed into a single mode fiber together with the other laser lines and all laser lines were coupled into a Yokogawa CSU-10 unit and focused onto the sample by a 100×, 1.49 NA objective. The fluorescence signal was split with the appropriate dichroics and focused onto 3 Andor iXon cameras equipped with the appropriate emission filters. A piezo element inserted into the stage enabled the recording of *z*-stacks and a heatable sample chamber allowed live cell measurements at 37 °C. A perfect focus system was used to compensate for *z*-drift during data acquisition.

Experiments were performed on a modified Andor Revolution system spinning disk confocal microscope (Andor Technology, Belfast, UK) (Figure A1). The system is built using a Nikon TE2000E (Nikon, Tokyo, Japan) microscopy body, a spinning-disk unit (CSU10; Yokogawa Electric Corporation, Musashino, Japan), an OptoSplit II (Cairn Research Ltd., Faversham, UK) for separating the eGFP and mCherry emissions and an EMCCD camera (DU-897 Ixon, Andor Technology, Belfast, UK) for detection. The excitation was controlled using an acousto-optic tunable filter (Gooch and Housego, Ilminster, UK) and the excitation and fluorescence emission were separated using a quadruple-band dichroic beam splitter (Di01-T405/488/568/647; Semrock, Rochester, NY, USA). The eGFP and mCherry signals were separated using a dichroic beamsplitter (BS562) and the respective emission filters (HC525/50, and ET605/70), all purchased from AHF Analysentechnik AG (Tübingen, Germany). *Z*-stacks were recorded over 20 min with an exposure time of 130 ms/frame/plane and 15–25 z positions spaced by 300 nm were acquired per *z*-stack. This resulted in a complete three-dimensional (3D) image every ~3 to 5 s.

### 2.3. Live-Cell Imaging Experiments

**Figure A2:**
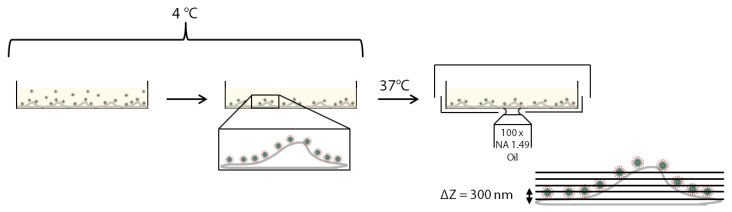
Sample preparation and data acquisition. Precooled HeLa cells were incubated double-labeled FV particles at 4 °C to allow membrane attachment and synchronize uptake. Immediately after transferring the sample onto the 37 °C warm microscope stage virus uptake was monitored by acquiring *z*-stacks of single cells decorated with not more than approx. 40 particles for a total time of about 20 min.

An overview of the live-cell imaging experiments is given in Figure A2. HeLa cells were cultivated in Dulbecco’s Modified Eagle Medium (DMEM) + 10% fetal bovine serum (FBS) at 37 °C in a 5% CO_2_ atmosphere and split every 2 to 3 days. Cells were seeded at 2 × 10^4^ cells per well in an 8-well Nunc LabTek II chambered coverglass slide coated with collagen A-solution (Sigma-Aldrich, St. Louis, MO, USA) according to the manufacturer’s protocol one day prior to experiments. On the day of the experiment, the cells were washed once with phosphate-buffered saline (PBS) solution and the virus particles were added at a density of 40 to 80 particles per cell in Leibovitz’s L15 medium containing 10% FBS. To allow attachment of the particles to the cell surface while avoiding virus uptake into the cells, cells were incubated with the virus at 4 °C for 10 min. Subsequently, the cells were rinsed with cold L15 medium and the imaging was started immediately after mounting the sample holder on the microscope stage and warming the cells to 37 °C to synchronize the uptake of the viruses.

Data was recorded over 20 min on single cells by acquiring multiple *z*-stacks spanning the entire cell volume with z-planes spaced 300 nm apart. EGFP and mCherry-labeled virus particles were excited in parallel with 488 nm and 561 nm continuous wave lasers. Figure A1 depicts the microscope setup used All cell culture reagents were purchased from Thermo Fisher Scientific, Waltham, Massachusetts, USA, if not stated differently.

### 2.4. Data Analysis

**Figure A3:**
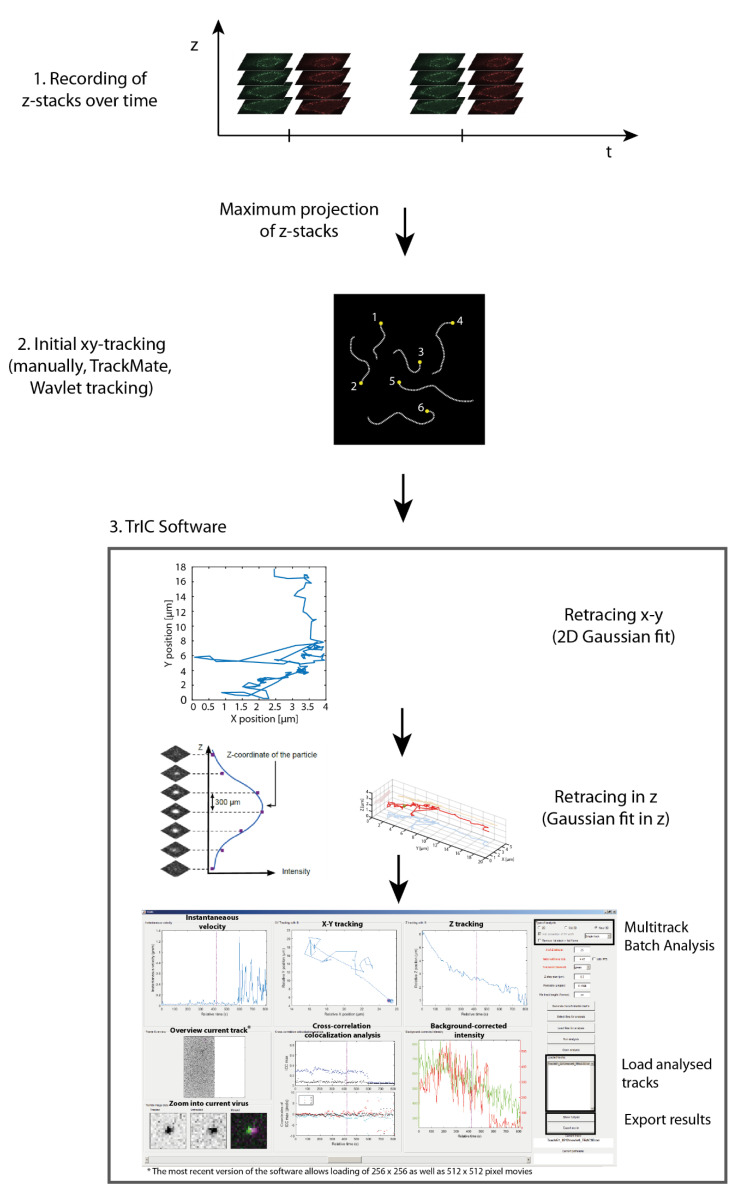
Data analysis via the TrIC software. Data was analyzed using the TrIC algorithm [30] in a home-written software in Matlab. The general workflow of the analysis and a screen shot of the data analysis program is shown. Starting in the top left, the program window shows: the instantaneous velocity, *x*-*y* projection of the trajectory, the z position as a function of time, frame overview, the image correlation analysis along the trajectory, a background-corrected fluorescence intensity of the two channels and images of the tracked and untracked channels and their overlay at the position of the cursor. Control parameters and functionalities in the program are given on the right.

The analysis was done using the TrIC software (Figure A3) previously developed in our lab [30]. Briefly, the virus is tracked in 3 dimensions in the eGFP channel. Single viruses are tracked in 2D either manually, automatically using TrackMate [34] or using a home-written wavelet tracking method [35]. The subpixel accurate 3D-trajectory is obtained in a second step by fitting the particle image with a 2D-gaussian function for the *x*-*y* position and a 1D-gaussian function for the *z* position. A box about the particle is taken (2.94 µm × 2.94 µm × ~5 µm or 21 pixels × 21 pixels × entire *z*-stack) and a 3-dimensional image cross-correlation is performed between the eGFP and mCherry channels. When a particle is detected in both channels, a positive correlation signal is observed. To determine a threshold for the amplitude of the correlation function, we also randomize the pixels in the voxel/box and perform the same analysis. Using the average value and standard deviation of the randomized signal from a sliding window of 10 3D-images, we define a threshold of the mean plus 3 standard deviations for the randomized image as the threshold for a positive cross-correlation signal. The software provides the background-corrected intensity of both fluorescence labels, subpixel accurate coordinates of the tracked virus in 3D for both detection channels, the instantaneous velocity of the particle, the 3D-colocalization status and the relative distance of the fluorescence labels along the track (Figure A3).

**Figure A4:**
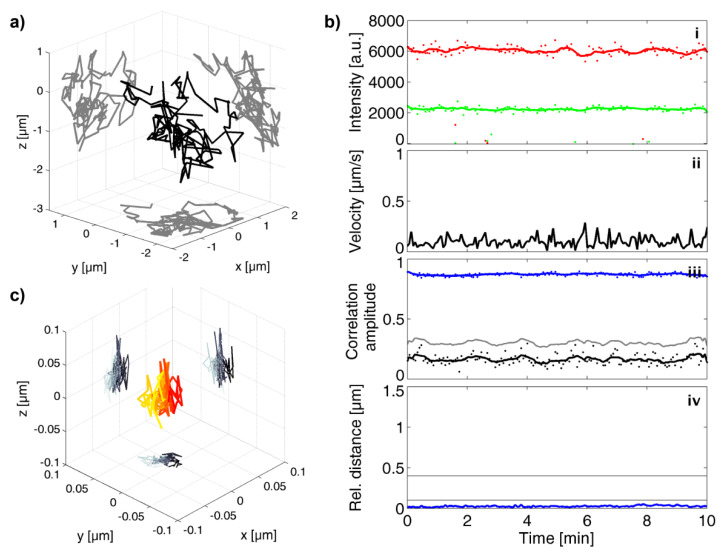
TrIC analysis on a dual-color fluorescent bead. As a positive control, we measured the diffusion of a dual-color fluorescent bead in a glycerol solution. (**a**) The 3D-trajectory and 2D-projections of a tracked bead. (**b**) The corresponding TrIC analysis: (**i**) The background-corrected fluorescence intensity of the green and the red signal are plotted as a function of time. (**ii**) The instantaneous velocity is plotted as a function of time. (**iii**) The amplitude of the cross-correlation of the TrIC analysis for the data (blue), the randomized control (black) and threshold (grey) are plotted as a function of time. (**iv**) The relative distance between the eGFP-capsid and mCherry-Env signal is plotted over time. (**c**). A 3D-representation of the relative position of the red signal with respect to the green signal color-coded as a function of time. The results show a high correlation amplitude and a relative distance of less than 100 nm over the whole trajectory.

To test the precision of our tracking method, we tracked dual-color fluorescent beads (TetraSpeck microspheres, 0.1 µm, Thermo Fisher Scientific, Waltham, MA, USA) in a glycerol solution (Figure A4). This trajectory shows the accuracy limit of our method. The mean relative distance was 25 nm over the ten minutes tracked with a standard deviation of 10 nm. For testing the resolution in the case of the dual-color viruses, we performed experiments on a fusion-deficient mutant, iFuse, and the mean relative distance was found to be 46 nm with a standard deviation of 20 nm, giving a 95% confidence boundary at 86 nm (data not shown). The threshold for separation of the two colors was then set to 100 nm.

### 2.5. Estimation of the Cell Surface

**Figure A5:**
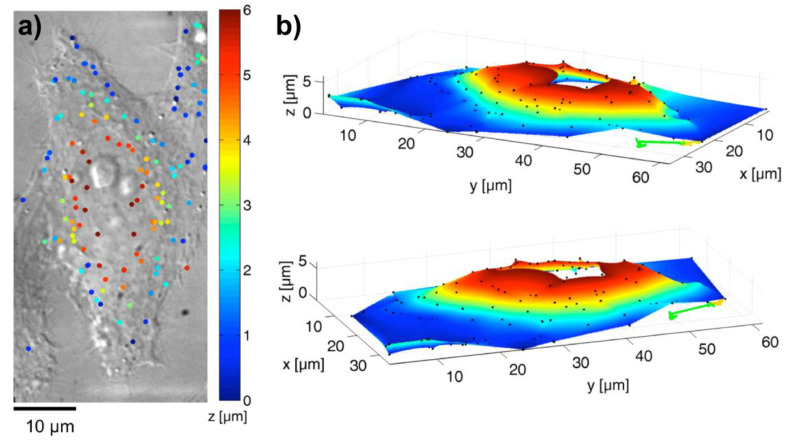
Estimation of the cell surface. The 3D-position of the virus particles was determined by 3D-Gaussian fitting on the first *z*-stack of the movie when the viruses are still bound to the plasma membrane of the cell. (**a**) An overlay of the virus particles’ positions with the DIC image of the cell. The *z*-position is color-coded. (**b**) The cell surface was reconstructed by interpolation of the virus’s positions and plotted as a 3D-surface together with the virus track (yellow and green after fusion).

As the cells were pre-incubated with the virus on ice in the refrigerator (~4 °C), most of the viruses are located at the cell membrane during the first frames of the movies. We took advantage of this situation to estimate the cell surface. The visible viruses were automatically detected in the first *z*-stack and their position was automatically determined in 3D via Gaussian fitting (see Figure A5a). The cell surface was then reconstructed from the single virus positions by a nearest neighbor interpolation and plotted as a 3D-surface together with a virus track to help determine the location of viral fusion with respect to the cell membrane (see Figure A5b).

## 3. Results

### 3.1. Characterization of Foamy Virus Particles

**Figure A6:**
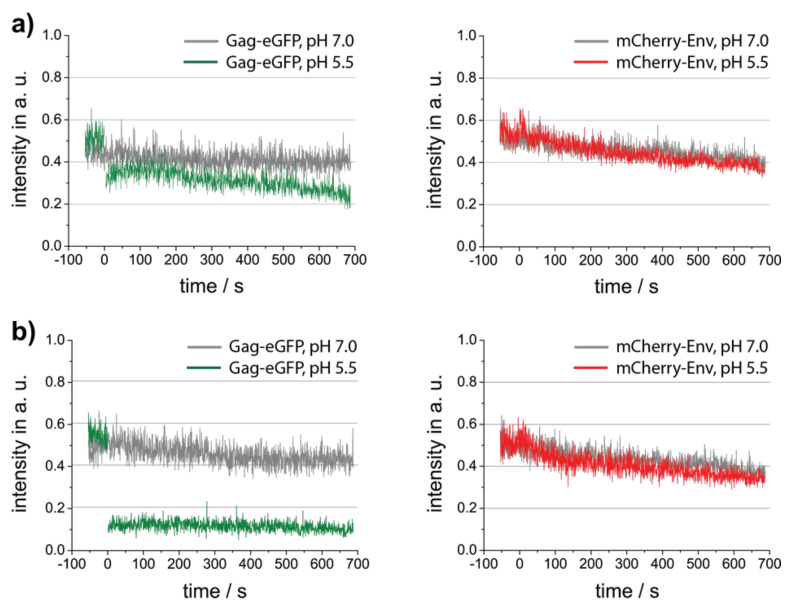
Stability of the fluorescent proteins on dual-color foamy virus particles at different pH values. Intensity traces of double-tagged PFV Env containing particles at pH 7.0 (gray) and pH 5.5 (green). At time point 0, citrate buffer was added to decrease the pH. The background-corrected intensities of ten particles were determined and the average is shown. The intensity of (**a**) intact particles and (**b**) control measurements on fixed and permeabilized particles measured on a coverslip are shwon. Note that the eGFP fluorescence showed a significant drop at pH 5.5.

With the aim of visualizing the fusion process of individual viruses, we further characterized the fluorescence-labeled FV particles that we designed previously [18]. Here, PFV and SFVmac Env-containing particles were labeled with eGFP and mCherry. It has been shown that endocytosis plays a significant role in the uptake of FV [18,19]. For endosomal uptake, the viruses encounter a decrease in pH from early endosomes (pH ~ 6.5), to late endosomes (pH ~ 5.5), to lysosomes (pH ~ 4.5). As it is known that eGFP fluorescence is quenched at acidic pH values [36], we tested the stability of the fluorescent proteins incorporated into the virus particles under these conditions (Figure A6). Particles labeled with eGFP attached to the Gag protein and mCherry attached to the Env protein were sedimented on a glass slide. The initial pH of 7.0 was then dropped to pH 5.5. A small reduction in the Gag-eGFP fluorescence upon lowering of the pH was observed, but the eGFP signal was still easily detectable (Figure A6a, left graph). mCherry-Env showed no change in fluorescence intensity (Figure A6a, right graph). For comparison, we first permeabilized the particles with Triton X-100 to allow access of the protons to the eGFP-labeled capsid. As expected, the eGFP fluorescence showed strong quenching at pH 5.5, a value typically found in endosomes (Figure A6b, left graph) whereas the mCherry fluorescence stayed unaffected (Figure A6b, right graph). Hence, the choice of label position with eGFP attached to the capsid works well even when the viruses have been taken up in endosomes. The eGFP is well shielded within the virus lipid envelope.

Secondly, a high fusogenicity is needed for these studies. Hence, as we have shown previously, we generated the viruses by mixing unlabeled Gag proteins with eGFP-tagged Gag at a ratio of 3:1 [18]. This mixture keeps the infectivity of the virus at near wild-type levels whereas labeling 100% of the Gag proteins with eGFP reduces the infectivity of the particles by approximately 90%. Labeling of the Env did not significantly alter the infectivity of the virus. Although a drop in infectivity, as determined by reporter gene expression, may not necessarily indicate a decrease in fusogenicity, a high infectivity does guarantee that the fusogenicity is also high. Thus, we used constructs using a mixture of 3:1 Gag:Gag-eGFP. From bulk infectivity assay and time-lapsed spinning disk confocal microscopy, we verified the fusogenicity of the viruses and could show that a significant fraction of PFV particles (~15%) underwent fusion during the first 30 min [18].

Lastly, to simplify detection of the fusion process, it is important that a high fraction of the prepared viral particles contain both labels. Fortunately, FVs cannot bud without the envelope protein [12,13]. Hence, 93 ± 1% of viral particles released into the cell culture supernatant were fluorescently labeled with Env-mCherry (Figure 1e,f). Conversely, about half of the viral envelopes were missing a detectible capsid signal [18]. This high number of presumably empty virus-like particles could be due to the optimized gene expression used to generate the virus [31,32], the known capability of FV to form sub-viral particles [17] and/or due to the attachment of a fluorescent protein to the N-terminus of a FV glycoprotein. Although a higher capsid incorporation would simplify the fusion measurements, 50% is the limitation of this viral system. However, with the high envelope labeling efficiency, the virus preparation is still well suited for investigating fusion using single virus tracing. A detailed characterization of the virus preparations is given in Table 1.

### 3.2. Investigation of Individual Prototype Foamy Virus Env-Mediated Fusion Events

To gain detailed insights into the fusion process, we performed live cell measurements using the fluorescently labeled FV particles described above. Cells were incubated with viral particles at ~4 °C. Subsequently, the cells were rinsed with cold L15 medium and the imaging was started immediately after warming the cells to 37 °C to synchronize the uptake of the viruses. Confocal *z*-stacks were collected over approximately 20 min and the data analyzed as outlined in the Section 2.

**Figure 2:**
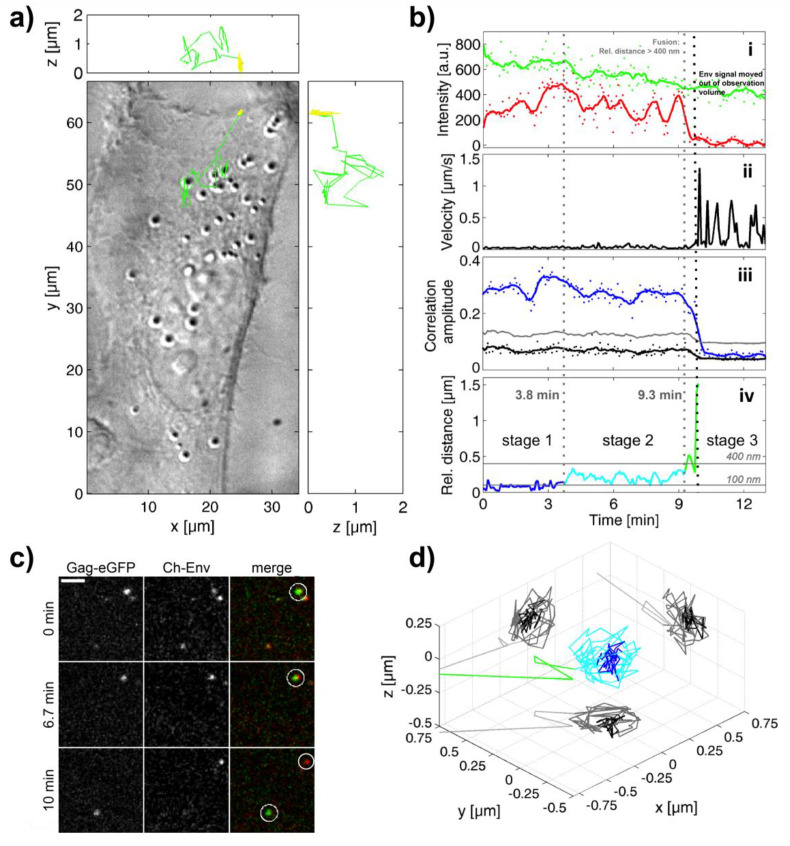
PFV Env-mediated fusion at the plasma membrane. (**a**) A differential interference contrast (DIC) image of a cell where the trajectory of the tracked PFV has been overlaid. The proportion of the track where the particle is double-labeled is plotted in yellow whereas movement of the capsid towards the nucleus/microtubule organizing center after color separation is plotted in green. *x-z* and *y-z* projects of the trajectory as shown in the upper and right panels respectively. (**b**) Results of the tracking imaging correlation (TrIC) analysis along the trajectory are shown: (**i**) The background-corrected fluorescence intensity of the Gag-eGFP channel (green) and the mCherry-Env channel (red) are plotted as a function of time. (**ii**) The instantaneous velocity of the Gag-eGFP signal is plotted as a function of time. (**iii**) The amplitude of the cross-correlation of the TrIC analysis for the data (blue), the randomized control (grey) and threshold (black) are plotted as a function of time. (**iv**) The TrIC analysis also provides the relative distance between the fluorescence signals in the two channels. The distance of the Gag-eGFP to the mCherry-Env is plotted over time, with distances <100 nm marked in blue (stage 1), between 100 and 400 nm marked in cyan (stage 2), and >400 nm marked in green (stage 3). Solid lines were generated using running average of ~30 s. (**c**) A close up of three frames from the movie showing the tracked virus (circled in white) at different stages of the fusion process: bound to the plasma membrane (0 min), during stage 2 (6.7 min), and after fusion (10 min) where the capsid has been transported within the cell. Left: eGFP channel; Middle: mCherry channel; Right: merged image. Scale bar: 4 µm. (**d**) A 3D-representation of the relative position of the mCherry-Env signal with respect to the Gag-eGFP signal distance color-coded according to the three stages shown in panel biv. The circular movement of the envelope signal around the capsid in stage 2 (cyan) is clearly visible.

Figure 2 and Movie S1 show the results of typical fusion of a PFV. Panel a of Figure 2 depicts the differential interference contrast (DIC) image of an infected cell overlaid with the trajectory of the infecting particle in three dimensions (3D). At the beginning of the movie, the virus is located at the plasma membrane. Both PFV Gag-eGFP and PFV mCherry-Env signals are observable, represented by the yellow color-coding in the trajectory. During the initial stage, the virus undergoes limited movement and slow photobleaching of the eGFP signal can be observed (Figure 2(bi)). Subsequently, the fluorescence signals begin to separate. At 9.7 min, the capsid is transported into the cytoplasm of the cell (green part of the trace) whereas the envelope remains on the plasma membrane (Figure 2a). To provide additional support that fusion actually occurred at the plasma membrane, we developed another method to help define the location of the plasma membrane. Before starting the acquisition of a movie, cells were incubated at low temperature to synchronize virus entry. As a consequence during the first *z*-stack, most viruses were still located on the cell surface. By automatically determining the 3D-position of all the fluorescent particles present in the first *z*-stack of the movie, the 3D-cell shape was inferred (see Materials and Methods, Figure A5). Comparison of the 3D-position of the tracked virus to the reconstructed 3D cell shape was then used to determine the location of the virus during the fusion. This method was helpful in identifying plasma fusion events but could also indicate fusion from a particle in the actin cortex just underneath the plasma membrane as the accuracy of the method is not sufficient to resolve these two cases.

The results of the TrIC analysis are shown in Figure 2b. The cross-correlation amplitude is a measure of the similarity of the image data in the two channels and a positive correlation above the control indicates a clear viral signal in both channels. At 9.7 min, the cross-correlation amplitude (Figure 2(biii)) between the two channels shows a clear drop indicating that the mCherry- Env signal is no longer in the box around the viral Gag-eGFP being tracked. This is a clear marker of color separation and indicates complete fusion of the virus. The drop in cross-correlation amplitude coincides with the loss of fluorescence intensity in the mCherry channel and with a sudden increase in the instantaneous velocity to values above 1 µm/s (Figure 2(bii)). The high velocity and directionality of motion indicates that the capsid is being actively transported towards the cell center. The transport velocity of 0.5 to 1 µm/s is consistent with values observed for direct transport of internalized viruses and endosomes along microtubules [23,24,37,38,39]. Thus, we assign the post-fusion active transport processes to the capsid hijacking cellular motor proteins and being transported along the microtubule network (summarized in [40]).

The image cross-correlation analysis also provides information about the relative distance between the signals in the two channels. As discussed in the materials and methods, we consider color separation to be significant when the relative distance increases over 100 nm. Figure 2(biv) depicts this information over the course of the observed fusion event. It can be subdivided into three stages. Initially, the separation between the eGFP and mCherry signals is approximately 100 nm or less (blue), which is within the detection limit. In stage 2, which starts about four minutes after the beginning of the track, the distance increases to values between 100 nm and 400 nm (cyan). This stage lasts for about 6 min. In stage 3 (green), separation rises above 400 nm until the mCherry-Env signal disappears from the tracking box around the Gag-eGFP signal, at which point the fusion is complete. Figure 2c shows single frames representative for the three stages from the recorded movie data. Plotting the relative distance of the envelope from the capsid signal in three dimensions (Figure 2d) reveals in stage 2 motion of the envelope around the capsid until the separation is completed.

### 3.3. Fusion Mediated by Simian Foamy Virus Env

**Figure 3:**
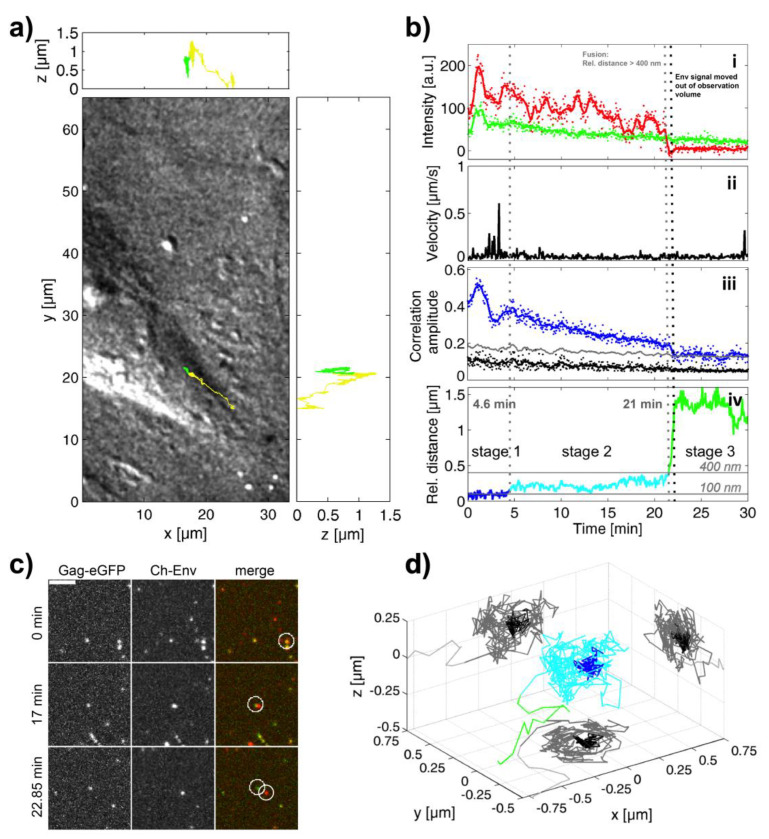
SFVmac Env-mediated fusion from the endosome. (**a**) A DIC image of a cell where the trajectory of the tracked SFVmac has been overlaid. The proportion of the track where the particle is double-labeled is plotted in yellow and motion of the capsid after color separation is plotted in green. *x-z* and *y-z* projects of the trajectory as shown in the upper and right panels respectively. (**b**) Results of the TrIC analysis along the trajectory are shown: (**i**) The background-corrected fluorescence intensity of the Gag-eGFP channel (green) and the mCherry-Env channel (red) are plotted as a function of time. (**ii**) The instantaneous velocity of the Gag-eGFP signal is plotted as a function of time. (**iii**) The amplitude of the cross-correlation of the TrIC analysis for the data (blue), the randomized control (grey) and threshold are plotted as a function of time. (**iv**) The relative distance between the eGFP-capsid and mCherry-Env signal is plotted over time. The distance of the Gag-eGFP to the mCherry-Env is plotted over time, with distances <100 nm marked in blue (stage 1), between 100 and 400 nm marked in cyan (stage 2), and >400 nm marked in green (stage 3). Solid lines were generated using running average of ~30 s. (**c**) A close up of three frames from the movie showing the tracked virus (circled in white) at different stages of the fusion process: bound to the plasma membrane (0 min), just after the virus has been internalized (17 min), and after the fusion is completed (22.85 min). Left: eGFP channel; Middle: mCherry channel; Right: merged image. Scale bar: 4 µm. (**d**) A 3D-representation of the relative position of the mCherry-Env signal with respect to the Gag-eGFP signal distance color-coded according to the three stages shown in panel (biv). The circular movement of the envelope signal around the capsid in stage 2 (cyan) is clearly visible.

As a comparison, we also investigated the fusion mediated by SFVmac Env (Figure 3 and Movie S2). For SFVmac Env, this viral particle was internalized, complete with envelope, within the first minute. The overlay of the 3D trajectory with the DIC image of the cell shows that the particle was actively transported towards the nucleus and that capsid release occurred internally (Figure 3a). Instantaneous velocities of up to 600 nm/s where measured (Figure 3(bii)). After 21 min, the particle fused with the surrounding cellular membrane (probably with an endosome) as indicated by a sudden drop of the mCherry-Env signal (Figure 3(bi)), a drop of the correlation amplitude (Figure 3(biii)) and an increase of the relative distance between the eGFP and mCherry signal to values above 400 nm (Figure 3(biv)). 

Again, for this event, the relative distance between the two signals can be divided into three stages (Figure 3(biv)): a stage in which the two signals colocalize within the detection limit of 100 nm (blue), an intermediate stage in which the relative distance varies between 100 and 400 nm (cyan) and finally full separation (green). Figure 3c shows three time points of the underlying image data highlighting the three stages. In the first two time points, there is at least some colocalization of the two signals. The lowest section shows the phase of full separation after ~22.3 min. We can also plot the 3D-distance between the eGFP labeled capsid and the mCherry-Env signal (Figure 3d). Here again, during the intermediate stage, the envelope and capsid signal move with respect to each other.

### 3.4. Analysis of All Observed Events

**Figure 4:**
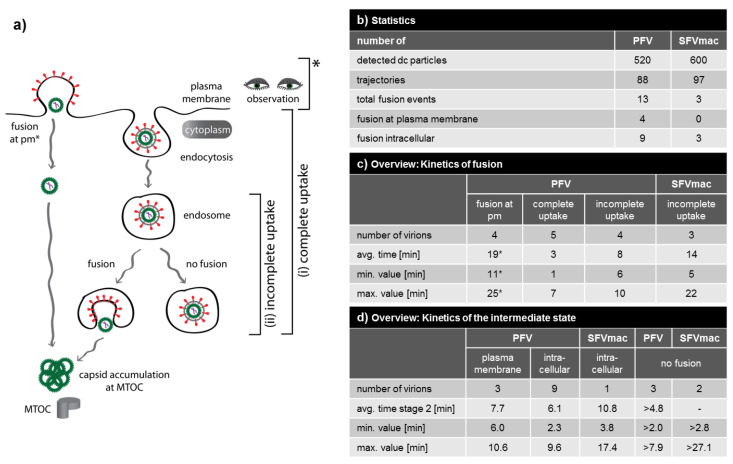
Overview of the detected fusion events. (**a**) Schematic illustration of the different types of fusions events detected during cellular uptake of dual-labeled FV particles. Events were classified into fusion at the plasma membrane and fusion from the endosome. Fusion events from an endosome were further divided into complete events (where the particle could be tracked from attachment at the plasma membrane until completion of the fusion from the endosome) and incomplete events for which the particle was only detected when it had already been taken up into an endosome. (**b**) Statistics of detected and tracked particles and detected fusion events. (**c**) Overview of the kinetics of fusion. * time at 37 °C until fusion. (**d**) Overview of the kinetics of the intermediate state.

We have demonstrated that it is possible to visualize fusion events using the fluorescently labeled FV particles harboring PFV Gag and PFV or SFVmac Env at different steps of viral uptake as schematically depicted in Figure 4a. Figure 4 gives a summary of all the observed single virus particles and fusion events observed for both types of FV particles. The number of fusion events detected was limited and thus the statistics are limited. From 520 detected PFV Env-containing particles, we tracked 88 individual virions and observed 13 fusion events (Figure 4b). Four fusion events were detected at the plasma membrane and nine events occurred from endosomes. The plasma membrane events were categorized as such when the particles were located on the plasma membrane (within the resolution of our microscope) and they have not undergone motion with velocities above 0.05 µm/s prior to fusion, consistent with transport velocities of particles transported on the plasma membrane [41,42]. The possibility of PFV Env being able to fuse at the plasma membrane is consistent with its ability to fuse to a significant extent already at neutral pH [18,19]. For fusion events categorized as occurring from endosomes, the particles demonstrated clear active transport before the fusion event was detected. In contrast to the plasma membrane fusion event shown in Figure 2 and Movie S1, the mCh-Env signal was typically lost within 5 to 15 s after the endosomal fusion event was completed, which is consistent with what has been reported for other viruses [24,43]. Disappearance of the mCh-Env signal is attributed to dilution of viral glycoproteins in host cell membranes after the fusion process and was observed both for fusion at the plasma membrane and in endosomes.

From the measurements, we can also gain insights into the kinetics of the fusion event. For fusion from the plasma membrane, the average time until fusion was 19 min. For fusion events from endosomes, it was necessary to separate the events into cases where the entire uptake was observed (Figure 4(ai)) and cases where the viruses had already undergone endocytosis before being detected (Figure 4(aii)). For the latter, it was not always possible to calculate the entire time until fusion. However, a minimum time could be estimated and, in either case, the time it took fusion to occur from the plasma membrane was significantly longer. The short lag time between entry until fusion in the case of endocytosed particles relative to fusion at the plasma membrane is in agreement with the reported pH-dependency of PFV fusion and the fact that early endosomes already have a slightly acidic pH around 6.5 to 6.0 [44,45,46]. In addition, early endosome fusions can be induced within 1 to 5 min after virus uptake (e.g., vesicular stomatitis virus, VSV), whereas late endosome fusion events (e.g., influenza A virus, INF) take longer, ranging from 10 to 20 min [44]. The kinetics of the fusion process are given in Figure 4c.

For SFVmac Env containing particles, we detected 600 viruses, tracked 97 particles, and detected three fusion events (Figure 4b). With these low statistics, it is not possible to make any definitive statements. What we did observe is that all three fusion events occurred from endosomes, consistent with the strong pH dependence of SFVmac Env-mediated fusion [18]. Although we can only estimate a minimum time until endosome fusion for SFVmac, it was significantly slower than that for endosomal fusion of PFV (Figure 4c). This is also consistent with the difference in uptake observed for PFV and SFVmac Env containing particles and may suggest that fusion of SFVmac occurs from late endosomes [18].

The PFV Env mediated uptake pathway has similarities with many other viruses. The uptake pathway resembles that of human immunodeficiency virus 1 (HIV-1) in that PFV infects target cells by fusion at the plasma membrane or by endocytosis [47]. PFV uptake also shows similarities with VSV, which fuses early after uptake in early endosomes [48]. In addition, the PFV fusion process can also be activated in maturating or late endosomes on the way towards the cell center as indicated by the fusion events observed in between 7 and 10 min after virus entry (Figure 4c). In contrast, we suggest that SFVmac Env containing particles fuse similar to INF viruses predominantly in late endosomal compartments [49]. The average minimum duration from uptake till fusion of 13.6 min supports this model.

### 3.5. A Puzzling Delay in Fusion

One of the surprising results of this investigation is the discovery of an intermediate step (stage 2) in the fusion process where the envelope and capsid signals are in close proximity, but are no-longer fully overlapping. Using the TrIC analysis, we detect a physical separation between the envelope and capsid signals of 100 to 400 nm. This is clearly greater than the accuracy of our tracking method. Both PFV and SFVmac Env-containing particles exhibited this intermediate step that was observed in all fusion events with the exception of one fusion event at the plasma membrane (Figure 4d). Occasionally, we observed viruses in the intermediate state that did not undergo complete fusion within the measurement time, but we never observed a virus return from the intermediate state into stage 1. Hence, the intermediate stage appears to be an important step in completing the fusion process in FV Env mediated entry, so we investigated it in more detail.

**Figure 5:**
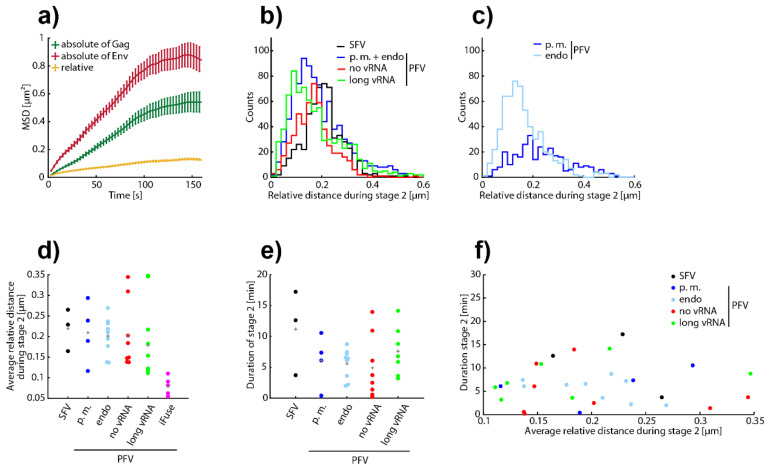
The intermediate stage during the fusion process. (**a**) A mean-square displacement (MSD) analysis of the absolute trajectory of the envelope signal (red), the absolute trajectory of the capsid (green) and of the relative trajectory of the envelope with respect to the capsid (yellow) during stage 2 of a SFVmac fusion event. The relative diffusion coefficient is clearly smaller than the absolute diffusion coefficients indicating that the two signals are tethered together. The Env protein diffuses faster than the Gag protein. Error bars show the standard error of the mean of the squared displacement. (**b**) Image-wise histograms of the relative distance of the capsid and envelope component within the intermediate step of the fusion process. PFV particles (blue) show a shift towards lower distances compared to SFVmac (black) or particles without a viral genome (red). (**c**) Image-wise histograms of the relative separation of envelope and capsid depending on whether fusion occurred from the plasma membrane (dark blue) or from endosomes (light blue). (**d**) A comparison of the average separation of the envelope from the capsid plotted per event for different types of FV particles. (**e**) A comparison of the average duration of the intermediate state per event for different types of FV particles. (**f**) A scatter plot of the average envelope-capsid separation versus duration of the intermediate fusion state for different types of FV particles. FV particle types: SFVmac virions at endosomes (SFV); PFV virions at the plasma membrane (PFV p.m.); PFV virions at endosomes (PFV endo); PFV virions that do not contain viral RNA (PFV no vRNA); PFV virions with longer viral RNA (PFV long vRNA); fusion incompetent PFV virions (PFV iFuse).

The first question we asked is what causes the two components to stay in close proximity. This could be caused by either spatial confinement, such as both components being trapped in an endosome or a pocket in the actin cortex, or the two components could be tethered together by an unknown component. To distinguish between these two alternatives, we compared the mean square displacement (MSD) analysis on the relative trajectory of envelope with respect to capsid and on the absolute trajectories of the Gag-eGFP and the mCh-Env during stage 2 (Figure 5a). If the two components are moving independently, the relative motion between them will be the sum of two random motions and will have a higher diffusion coefficient than the absolute diffusion coefficients. On the other hand, if the two components are tethered together, the relative diffusion coefficient will be less than that of the absolute diffusion coefficients. The early times need to be compared, as confinement in an endosome will limit the sensitivity of the MSD analysis to the differences in motion. Figure 5a shows a MSD analysis of the absolute diffusion of the Gag-eGFP and mCh-Env in comparison to the relative motion for an SFVmac Env fusion event from an endosome. The relative diffusion coefficient is clearly smaller than the absolute diffusion coefficients, suggesting that the two components are tethered together. This is true for all fusion events measured.

Next, we investigated the average separation and duration of the intermediate state (stage 2) for the different categories of fusion events. Image-wise histograms of the envelope-capsid separation is plotted in Figure 5b and shows a peak with lower average separation for PFV Env constructs (blue, green, red) in comparison to SFVmac Env (black). A small difference is also observable when investigating the average separation per event (Figure 5d). When separating the PFV Env events into fusion from the plasma membrane and from endosomes, the image-wise histograms show a narrow peak in the separation for endosomal fusion events compared to a broader histogram for fusion from the plasma membrane (Figure 5c). Although the statistics are too low to make a significant statement, this could be due to confinement in endosomes limiting the maximum possible separation. The fusion events from endosomes for SFVmac Env (Figure 5b, black) exhibit an average larger separation then the endosomal fusion events measured for PFV Env (Figure 5b–d, black versus light blue), which would suggest that fusion occurs from different types of endosomes. When investigating the separation and duration of the intermediate state (Figure 5d–f), no significant trend is observable. The average duration of stage 2 in SFVmac Env mediated fusion events is longer, but this can be attributed to one very long event out of three. Hence, the statistics are not sufficient to make any significant statements here. Moreover, the scatterplot of duration versus average separation shows no correlation (Figure 5f).

Furthermore, we considered what could be physically tethering the envelope and capsid together over hundreds of nanometers. One possible candidate would be the viral genome, although one would expect the genome to be packaged inside the capsid during fusion and not having physical contact with the Env protein. We prepared several variants of PFV Env containing particles with differences in the packed genome (Figure 1e,g and Table 1). The first variant (no vRNA) were particles containing wild type PFV Gag-eGFP but no viral genome was co-expressed during virus productions. In this case, the distance distribution and duration of the intermediate state was not significantly different for SFVmac Env (SFV) or for endosomal fusion from PFV Env containing particles (PFV endo) with regular PFV genomes (Figure 5d,e). However, this PFV variant, though it does not contain a viral genome, still packages nonspecific cellular mRNA [33]. As a next step, we generated a PFV variant (iNAB) that was incapable of binding and incorporating any kind of RNA due to deletion of the PFV Gag nucleic acid binding domain [33]. However, we were unable to detect any fusion events for this construct (data not shown). For the final variant (long vRNA), we replaced for virus production the regular transfer vector expressing the encapsidated viral RNA genome by a transfer vector expressing a viral RNA genome that had been extended by 1319 bases, or an increase of 20% (Figure 1e). For this PFV variant, we saw no significant difference in the average separation or the duration of the fusion event (Figure 5e). Details of the intermediate state are summarized in Figure 4d.

## 4. Discussion

Using the recently developed dual-colored PFV virions containing Gag-eGFP and mCherry-Env labels, we were capable of detecting individual fusion events. Strikingly, in 185 viral particle tracks, we were able to observe a total of 16 fusion events, characterized by a loss of colocalization and separation of Gag-eGFP and mCherry Env signals. This is in contrast to a previous study for HIV-1 by Koch and colleagues who used an ecotropic murine leukemia virus (eMLV) Env-YFP combined with HIV-1 MA-mCherry to study particle fusion [50]. From more than 20,000 2D trajectories, they were able to detect 28 fusion events of rapid color separation. In addition, the authors identified 45 events of simultaneous loss of MA and Env signal, which is most likely due to endocytosis of double-labeled particles. Simultaneous disappearance of MA and Env signal was also observed for particles pseudotyped with fusion-deficient Env proteins, although to a lower extent. Hence, FV is much more fusogenic than eMLV pseudotyped HIV-1.

In our study, we detected for PFV Env containing particles fusion both at the plasma membrane as well as from endosomes whereas fusion of SFVmac Env containing particles was only detected from endosomes. This is in line with previous results demonstrating that Env protein of PFV is the only FV Env examined so far that already has a significant fusion activity at neutral pH, thereby enabling fusion at the plasma membrane of the host cells [18,19].

However, even PFV Env mediated fusion is known to be enhanced by low pH [19]. In agreement with this, we also observed PFV Env-mediated fusion events after endocytic uptake of viral particles. By comparing the kinetics of viral fusion after endocytic uptake between PFV Env- and SFVmac Env containing particles, we found that the time from uptake to fusion was also faster for PFV Env in comparison to SFVmac Env containing particles. This may suggest that pH-triggered PFV Env mediated fusion takes place in a different endosomal compartment with higher pH, perhaps in early endosomes, whereas SFVmac Env mediated fusion may only be triggered by the lower pH found in late endosomes. However, further single particle tracking studies including fluorescent markers for endosomal subpopulations are required to verify this hypothesis.

Using the TrIC approach, we detected a novel intermediate state during the fusion process where the envelope and capsid separate on the range of 200 to 400 nm that occurs for 6 to 10 min before complete fusion occurs. During this intermediate state, the envelope and capsid are still tethered. We showed that the fusion intermediate is not just due to co-confinement and could rule out that it is the viral genome that tethers the envelope and capsid together during this intermediate state. Fusion of membrane enveloped virus particles with host cell membranes is a multi-step process involving membrane merging via a universal “cast-and-fold” mechanism. Fusion-protein-mediated membrane merging is characterized by different intermediate structures including stalk formation, hemifusion, pore formation, pore growth, and, finally, capsid delivery [51,52]. It may be possible that the ability to detect the intermediate state during fusion of FV Env is dependent on the location of the fluorescent tag on the FV glycoprotein. All glycoproteins used in this study had the fluorescent protein tag fused to the cytoplasmic N-terminus of the Env LP-subunit, which adopts a type II membrane topology (Figure 1a). LP is thought to be an integral component of the heterotrimeric FV glycoprotein complex, also containing SU- and TM subunits [10]. Structural glycoprotein complex rearrangements in the multi-step fusion process may lead to release of LP from the other subunits during early phases and a higher mobility of the free, tagged LP subunit within the surrounding membrane(s) resulting in a greater distance to the tagged FV capsid. Alternatively, or in addition, the unique organization of trimeric PFV glycoprotein complexes in intertwined hexagonal networks on the surface of virions [8,10] may be responsible for the occurrence of the intermediate state. Perhaps expansion of the FV Env-mediated fusion pore, which is required for release of the capsid into the cytoplasm, is progressing slower than for other retroviral glycoproteins because the transmembrane helices of gp18^LP^ and gp48^TM^ may move as a block during fusion as suggested by Effantin and colleagues [8,10]. This may enable detection of an intermediate state with an increased distance between Gag and LP labels and their tethering for a certain time period.

In future studies, it would therefore be interesting to identify functional FV Env variants with fluorescent tags in the SU- or TM subunit and determine whether fusion events of such dual-colored FV virions also allow identification of an intermediate stage 2 fusion state. Furthermore, placing this fusion intermediate state in context with the other steps in the fusion process and to determine the actual biomolecules responsible for tethering the capsid and Env proteins during the fusion process, is of great interest for follow-up studies.

## Figures and Tables

**Table 1:** Characteristics of dual-color labeled samples.

Label of Virus Prep	vgRNA	Viral Gag	Viral Env	Gag Ratio Tagged: Untagged	∑ Green Particles	% Coloc. Green ± SEM	∑ Red Particles	% Coloc. Red ± SEM
PFV wt ^1,2^	wt	PG EG	PE Ch	1:0	6666	97 ± 0.8	12,649	55 ± 0.5
PFV wt ^2^	wt	PG EG	PE Ch	1:3	2365	98 ± 0.2	7683	33 ± 0.7
PFV wt ^1,2^	wt	PG EG	PE Ch	1:3	1476	93 ± 1	4022	33 ± 1
PFV iFuse ^1^	wt	PG EG	PE Ch iCS1	1:3	542	95 ± 1	1159	48 ± 2
PFV no vRNA	-	PG EG	PE Ch	1:3	168	100 ± 0	351	48 ± 6
PFV long vRNA	long	PG EG	PE Ch	1:3	152	94 ± 6	235	64 ± 5
SFV wt ^1^	wt	PG EG	SE Ch	1:3	944	93 ± 0.8	3290	30 ± 1

^1^ Characterization of these particle lots was already described in [18]. ^2^ Different lots of dual-colored, wildtype PFV particles.

## Supplementary Materials

The following are available online at https://www.mdpi.com/1999-4915/12/12/1472/s1, Movie S1: Fusion of PFV at the plasma membrane; Movie S2: Fusion of SFVmac from an endosome.

## Abbreviations

DIC	differential interference contrast
DMEM	Dulbecco’s Modified Eagle Medium
eGFP	enhanced green fluorescent protein
Env	envelope
eMLV	ecotropic murine leukemia virus
FBS	fetal bovine serum
FV	foamy virus
Gag	group specific antigen
HIV-1	human immunodeficiency virus 1
INF	influenza A virus
LP	leader peptide
mRNA	messenger RNA
MSD	mean square displacement
PBS	phosphate-buffered saline
PFV	prototype foamy virus
Pol	polymerase
RNA	ribonucleic acid
SFVmac	macaque simian foamy virus
SFVmcy	simian foamy virus Macaca cyclopis
SFVpsc	simian foamy virus Pan troglodytes schweinfurthii
SU	surface
SVT	single virus tracing
TM	transmembrane
TrIC	tracking image correlation
vRNA	viral RNA
VSV	vesicular stomatitis virus
YFP	yellow fluorescent protein
