# Supplementary material for: Identification of an Intermediate Step in Foamy Virus Fusion
Source: Viruses. 2020 Dec 21;12(12):1472. doi: 10.3390/v12121472 (PMC7766700; doi:10.3390/v12121472)
Supplement: Supplementary file 1 [file viruses-12-01472-s001.zip › Supplementary Material/Supplementary Material 201019.pdf]

Supplementary Movie S1: Fusion of PFV at the plasma membrane.

This movie shows the results of the fusion event from Figure 2. The upper left panel shows images from the Gag-eGFP channel, used for tracking the capsid, the middle panel shows the mCherry-Env channel, indicating the presence of the envelope and the right panel shows the merged image (eGFP signal in green, mCherry signal in red). The field of view corresponds to 20 μm x 26.81 μm. The lower left panels show the results of the TrIC analysis with the total intensity of the two channels along the trace. The top panel shows the background-corrected fluorescence intensity of the Gag-eGFP channel (green) and the mCherry-Env channel (red) as a function of time. The middle panels shows the amplitude of the cross-correlation of the TrIC analysis for the data (blue), the randomized control (grey) and threshold are plotted as a function of time. The lower panel shows the relative distance between the eGFP-capsid and mCherry-Env signal as a function of time. The cursor shows the position of the given timepoint plotted in the movie. Three stages are observable: Stage 1 (blue) where the distance is smaller than 100 nm, which is roughly the tracking accuracy. Stage 2 (cyan) where the distance increases between 100 to 400 nm (cyan). In stage 3 (green), the distance rises to values above 400 nm and the envelope signal moves out of the observation volume indicating completion of the fusion event. The right panel shows the relative position of the mCherry-Env signal with respect to the Gag-eGFP signal color-coded according to the three stages shown in the lowest left panel. The circular movement of the envelope signal around the capsid in stage 2 is clearly visible.

Supplementary Movie S2: Fusion of SFVmac from an endosome.

This movie shows the results of the fusion event from Figure 3. The upper left panel shows images from the Gag-eGFP channel, used for tracking the capsid, the middle panel shows the mCherry-Env channel, indicating the presence of the envelope and the right panel shows the merged image (eGFP signal in green, mCherry signal in red). The field of view corresponds to 17.75 μm x 18 μm. The lower left panels show the results of the TrIC analysis with the total intensity of the two channels along the trace. The top panel shows the background-corrected fluorescence intensity of the Gag-eGFP channel (green) and the mCherry-Env channel (red) as a function of time. The middle panels shows the amplitude of the cross-correlation of the TrIC analysis for the data (blue), the randomized control (grey) and threshold are plotted as a function of time. The lower panel shows the relative distance between the eGFP-capsid and mCherry-Env signal as a function of time. The cursor shows the position of the given timepoint plotted in the movie. Three stages are observable: Stage 1 (blue) where the distance is smaller than 100 nm, which is roughly the tracking accuracy. Stage 2 (cyan) where the distance increases between 100 to 400 nm (cyan). In stage 3 (green), the distance rises to values above 400 nm and the envelope signal moves out of the observation volume indicating completion of the fusion event. The right panel shows the relative position of the mCherry-Env signal with respect to the Gag-eGFP signal color-coded according to the three stages shown in the lowest left panel. The circular movement of the envelope signal around the capsid in stage 2 is clearly visible.
